# Integrated Metabolome and Transcriptome Analyses Reveal the Efficacy of Steroidal Saponins for Glucose and Lipid Metabolism in Hybrid Grouper (♀*Epinephelus fuscoguttatus* × ♂*Epinephelus lanceolatu*) Fed Higher-Lipid Diets

**DOI:** 10.3390/ani13182894

**Published:** 2023-09-12

**Authors:** Hongjin Deng, Guiqiong Chen, Jiacheng Zhang, Qihui Yang, Xiaohui Dong, Shiwei Xie, Weixing Liang, Beiping Tan, Shuyan Chi

**Affiliations:** 1 Laboratory of Aquatic Nutrition and Feed, College of Fisheries, Guangdong Ocean University, Zhanjiang 524088, China; denghongjin1020@163.com (H.D.); zhangjiacheng68@163.com (J.Z.); qihuiyang03@163.com (Q.Y.); dongxiaohui2003@163.com (X.D.); xswzsdx@163.com (S.X.); lwx13435981133@126.com (W.L.); bptan@126.com (B.T.); 2 Guangzhou Fishtech Biotechnology Co., Ltd., Guangzhou 510640, China; ja0ja@163.com; 3 Guangdong Engineering Technology Research Center of Aquatic Animals Precision Nutrition and High Efficiency Feed, Zhanjiang 524088, China

**Keywords:** transcriptome, metabolome, steroidal saponins, glucose and lipid metabolism, Serranidae, higher-lipid diets

## Abstract

**Simple Summary:**

The ingestion of higher-lipid diets by fish could lead to excessive lipid deposition and metabolic disorders in vivo. This experiment analyzed the effects of steroidal saponins on hepatic glucose and lipid metabolism in hybrid grouper fed higher-lipid diets using a multi-omics approach. Data from grouper fed diets with 0% and 0.1% steroidal saponins show that 0.1% steroidal saponins inhibited glycogen synthesis, gluconeogenesis, and the arachidonic acid metabolism pathway and activated glycogenolysis, glycolysis, and the fatty acid β-oxidation pathway. This study provides a deeper insight into the glycolipid metabolic processes in the liver of grouper fed higher crude-lipid diets.

**Abstract:**

An analysis of the extent of the effect of steroidal saponin addition on glucose and lipid metabolism in hybrid grouper liver was performed at the transcriptomic and metabolomic levels. Feeds (52% crude protein, 14% crude lipid) were prepared containing 0% (S_0_), 0.1% (S_0.1_), and 0.2% (S_0.2_) steroidal saponins. After eight weeks of feeding trial, compared to the S_0_ group, the activities of serum albumin, alanine aminotransferase, and aspartate transaminase were significantly lower and the activities of lysozyme, acid phosphatase, and alkaline phosphatase were significantly higher in the S_0.1_ group (*p* < 0.05). The superoxide dismutase, catalase, and glutathione peroxidase activities in the livers of the S_0.1_ group were significantly higher than those of the S_0_ group, while the malondialdehyde content was significantly lower than that of the S_0_ group (*p* < 0.05). There were forty-two differentially expressed genes and thirty-two differential metabolites associated with glucose and lipid metabolism enriched using KEGG and GO. In the S_0_ group, the expression of prostaglandin-endoperoxide synthase 1, prostaglandin E synthase 1, and thromboxane-2 synthase mRNA was significantly higher than in the S_0.1_ group (*p* < 0.05). The expression levels of genes in the S_0_ group were significantly higher than those in the S_0.1_ group (*p* < 0.05), including for glycogen synthase kinase, glucose-6-phosphatase catalytic subunit 2, fructose-1,6-bisphosphatase, phosphoenolpyruvate carboxykinase, glucose transporter 4, and malate dehydrogenase. The expression of mRNA such as fatty acid synthase, acetyl-CoA carboxylase, and sterol regulatory element-binding protein 1 was significantly lower in the S_0.1_ group than in the S_0_ group, while the expression of carnitine acyltransferase 1, acyl-CoA synthetase, and acyl-CoA dehydrogenase genes was significantly higher in the S_0_ group (*p* < 0.05). In summary, glycogen synthesis, gluconeogenesis, and the arachidonic acid metabolism pathway were inhibited by 0.1% steroidal saponins, and glycogenolysis, glycolysis, the tricarboxylic acid cycle, and the fatty acid β-oxidation pathway were activated. This study aims to provide a reference for the formulation of grouper feeds with a higher crude-lipid level.

## 1. Introduction

In the past five years, China’s cultivated grouper production grew from 131,500 tons to 204,100 tons, an increase of 55.21% [1,2]. The development of compound diet technology is a key factor in ensuring grouper production. Further, fishmeal, which is an optimal protein source for grouper diets, is rich and balanced in nutrients, especially in protein and fatty acids [3]. Between 2017 and 2021, Chinese fishmeal production rose from 639,200 tons to 659,000 tons. However, in fact, the annual demand for fishmeal in China was about 1.7 million tons, which meant that the Chinese fishmeal imports had increased from 1,571,600 tons to 1,823,400 tons with an external dependence of over 70% [1,2,4,5]. In view of this, fishmeal influenced the formula composition and price of feed and limited the efficient development of aquaculture.

Appropriately increasing lipid content in diets is an effective measure to improve protein efficiency and feed utilization [6,7,8,9], but long-term intake of high-lipid diets can result in abnormal accumulation of hepatic lipids and induce metabolic diseases such as fatty liver, which leads to metabolic disorders and immune suppression in the organism, affecting fish growth and reducing the economic efficiency of culturing [10,11].

The *Panax notoginseng* saponin can enhance the antioxidant capacity of the organism by increasing superoxide dismutase and catalase enzyme activities and decreasing malondialdehyde content in the liver of grouper [12]. *Yucca schidigera* saponins and soya saponins were found to reduce malondialdehyde, total cholesterol, and triglyceride levels in the serum and liver of white shrimp (*Litopenaeus vannamei*) and turbot (*Scophthalmus maximus* L.) [13,14]. The steroid saponins belong to a steroidal class of saponins, including spirostanes, furanosteroids, and furanospiralosteroids, which have the ability to scavenge free radicals, such as -OH and O^2−^ [15], to lower serum and hepatic levels of cholesterol and triglycerides [16] as well as the ability to promote the breakdown of hepatic glycogen [17]. The bitter melon saponins (*Momordica charantia*, steroids) have been shown to enhance glucose metabolism by preventing the absorption of glucose in the small intestine of mice and promoting the breakdown of glycogen in the liver [18,19]. Saikosaponin d has been confirmed to alleviate high-fat-diet-induced hepatic steatosis in hybrid grouper by targeting the AMPK/PPARα pathway [20]. Our previous study found that steroidal saponins improve the utilization of lipids and glucose in grouper liver when the fish ingested a high-lipid diet [21]. What is the exact mechanism?

The transcriptome is the pivot that links the genetic information of genes to functional proteins [22]. For example, it can explain that taurine can promote hepatic endogenous taurine synthesis, bile secretion, insulin secretion, and fatty acid β-oxidation efficiency to reduce fat accumulation in grouper ingesting high-lipid diets (15% crude lipid) [23]. As a bridge between genotype and phenotype, the metabolome ensures the maintenance of aerobic metabolism and activates gluconeogenesis as key metabolic strategies of turbot kidney against heat stress [24]. Association analysis of transcriptomic and metabolomic data provides a more detailed analysis of the relationship between critical genes and metabolites to explore the potential mechanisms of the organism [25]. The down-regulated key genes (lysophosphatidylcholine acyltransferase and glycerol-3-phosphate dehydrogenase) and increased metabolites (choline and taurine) were found to contribute to the alleviation of lipid metabolism disorders caused by elevated temperature stress in turbot [26]. In this study, we combine transcriptome and metabolome association analysis to investigate how steroidal saponins alter hepatic glycolipid metabolism in grouper.

## 2. Materials and Methods

### 2.1. Experimental Design

Three isoproteic (52% crude protein) and isolipidic (14% crude lipid) diets were formed by adding 0% (S_0_), 0.1% (S_0.1_), and 0.2% (S_0.2_) steroidal saponins (Guangzhou Fishtech Biotechnology Co., Ltd., Guangzhou, China) to diets with fishmeal, concentrated cottonseed protein, poultry by-product meal, and soybean meal as the main protein source and fish oil and soybean oil as the main lipid sources. Please refer to Supplementary Material (Tables S1 and S2). Experimental fish were fed the experimental diets to apparent satiation at 8:00 and 16:00 daily for 8 weeks. The hybrid grouper were separated into nine buckets (1 m^3^ Fiberglass farming buckets) with twenty-five fish in each bucket. During the experiment, the fish were continuously oxygenated every day, dissolved oxygen 5–6 mg/L, ammonia nitrogen <0.2 mg/L, temperature 30.5 ± 0.8 °C, salinity 28–32. Please refer to our previous study for the detailed feeding process [21].

### 2.2. Sample Collection and Chemical Analysis

Blood samples were obtained from the tail vein of hybrid grouper. The samples were centrifuged at 4 °C and 3500 rpm for 15 min to extract the serum and then immediately stored at −80 °C.

Meanwhile, the soybean-sized liver (approximately 600 mg) was obtained from each replicate on ice and then washed with saline. At once, the liver samples were stored at −80 °C for analysis of the gene expression of glucose lipid metabolism enzyme.

The grouper were anesthetized with MS-222 (1:10,000), and then the fresh soybean-sized liver was cut from living fish fed diets S_0_ and S_0.1_ and immediately washed with saline to clean the surface of blood remaining on the tissue [21]. The liver samples were numbered, stored in 2 mL freeze tubes, quickly put in liquid nitrogen, and subsequently transferred to −80 °C for metabolome and transcriptome analyses. The detailed methodology was described in Yang and Li et al. [9,13].

Serum parameters were measured using commercial kits (Nanjing Jiancheng Institute of Biological Engineering Co., Ltd., Nanjing, China). The parameters included total protein (TP, microplate method, A045-4-1), albumin (ALB, microplate method, A028-2-1), alanine aminotransferase (ALT, microplate method, C009-2-1), aspartate transaminase (AST, microplate method, C010-2-1), lysozyme (LZM, turbidimetric method, A050-1-1), acid phosphatase (ACP, microplate method, A060-2-2), and alkaline phosphatase (AKP, microplate method, A059-2-2).

Antioxidative enzymes in liver including superoxide dismutase (SOD, hydroxylamine method, A001-2-2), catalase (CAT, ammonium molybdate method, A007-1-1), glutathione peroxidase (GSH-PX, colorimetric method, A005-1-2), and hepatic malondialdehyde (MDA, TBA method, A003-1-2) contents were analyzed with commercial kits (Nanjing Jiancheng Institute of Biological Engineering Co., Ltd., Nanjing, China).

### 2.3. Metabolome Analysis

The 100 mg liver samples (six replicates per group) were ground separately in liquid nitrogen, and 500 μL of precooled 80% methanol was resuspended using well vortex to homogenize. The liver samples were ice-bathed for 5 min, followed by centrifugation at 15,000× *g* for 20 min at 4 °C. The supernatants were diluted with mass-spectrometry-grade water to a methanol content of 53% and then centrifuged (15,000× *g*, 20 min, 4 °C). Thus, the supernatants were injected into the UPLC–MS/MS system for analysis. UPLC–MS/MS analyses were conducted at Gene Denovo Ltd. (Guangzhou, China), and a Vanquish UPLC system (ThermoFisher, Berlin, Germany) and an Orbitrap Q ExactiveTM HF-X mass spectrometer (Thermo Fisher, Berlin, Germany) were used.

The raw data files were processed using UPLC–MS/MS with Compound Discoverer 3.1 (CD3.1, Thermo Fisher) performing peak alignment, peak extraction, and quantification for each metabolite. Peak was matched to mzCloud (https://www.mzcloud.org/, accessed on 16 February 2022), mz Vault, and Mass List database to obtain accurate qualitative and relative quantitative results. We used the statistical software R (R-3.4.3 version), Python (Python version 2.7.6), and CentOS (CentOS version 6.6) for statistical analysis. A normal transformation was attempted using the area normalization method when the data were not normally distributed.

### 2.4. Transcriptome Analysis

The total RNA of liver (about 300 mg) was extracted with Trizol reagent (Invitrogen, Carlsbad, CA, USA) according to the manufacturer’s protocol. The RNA quality integrity and concentration were assessed with agarose gel electrophoresis and Nanodrop 2000 assay. The input concentration of RNA sample per replicate in S_0_ group was 765.8, 576.9, and 406.3 ng/uL; in S_0.1_ group, 764.7, 537.0, and 792.5 ng/uL; in S_0.2_ group, 808.7, 675.4, and 855.0 ng/uL. The enriched mRNA was then fragmented with fragmentation buffer and reversed to cDNA using the NEBNext Ultra RNA Library Prep Kit for Illumina (NEB #7530, New England Biolabs, Ipswich, MA, USA). The cDNA libraries were sequenced with Gene Denovo Biotechnology (Guangzhou, China) using Illumina Novaseq6000.

The raw reads were quality controlled using fastp (https://github.com/OpenGene/fastp, accessed on 14 February 2022) software with default parameters to obtain clean reads. All assembled sequences were matched to the protein sequence databases Kyoto Encyclopedia of Genes and Genomes (KEGG, http://www.kegg.jp/, accessed on 14 February 2022) and Gene and Genomes Database (GO, http://geneontology.org, accessed on 14 February 2022) with blastx (E-value cut-off of 8 × 10^5^). The following differential expression analysis and functional enrichment were performed on the Omicsmart cloud platform (https://www.omicsmart.com, accessed on 14 February 2022). The expression of each unigene was calculated using the RSEM program (http://deweylab.github.io/RSEM/, accessed on 14 February 2022). Differentially expressed unigenes (DEGs) were analyzed using DESeq2 software with |log_2_FC| > 1 and *p* value ≤ 0.05 (http://www.bioconductor.org/packages/release/bioc/html/DESeq.html, accessed on 14 February 2022).

### 2.5. Quantitative RT-PCR Analysis of Gene

Quantitative reverse transcription PCR (qRT-PCR) was performed to verify the relative expression levels of the fourteen selected DEGs so that the gene expression data obtained with RNA-Seq could be validated. Liver total RNA was extracted with Trizol reagent (Invitrogen, Carlsbad, CA, USA). The cDNA was composed with the Prime Script RT kit (Takara, Osaka, Japan), and the quantitative thermal cycler (Light Cycler 480II, Roche Diagnostics, Basel, Switzerland) was used for qRT-PCR with the SYBR Premix Ex Taq kit (Takara, Osaka, Japan) within a reaction volume of 10 μL, including 3.2 μL of sterilized double-distilled water, 1 μL of cDNA, 0.4 μL of each primer, and 5 μL of SYBR Premix Ex Taq (Takara, Osaka-shi, Japan). Requirements were a cycle of 30 s at 95 °C followed by thirty-five cycles at 95 °C for 5 s, 60 °C for 25 s, and 72 °C for 30 s. The β-actin (AY510710.2) was used as internal control. The results were calculated using the 2^−ΔΔCt^ method [12]. A list of primers is given in Table 1.

### 2.6. Statistical Analysis

Mean ± standard error (SE) was used to present the results of the experiment. The statistic was analyzed using one-way analysis of variance (ANOVA) followed by Tukey’s multiple comparison tests with the software SPSS 21.0 (SPSS Inc., Michigan Avenue, Chicago, IL, USA). Significant differences were identified as *p* < 0.05. The Graph Pad Prism 8.0 software (8.0.2.263) was used to image experimental results.

## 3. Results

### 3.1. Activities of ALT, AST, and Levels of TP, ALB in Serum

The results of the serum biochemical indexes are shown in Table 2. The enzyme activity of ALT between the S_0.1_ and S_0.2_ groups was not significantly different, but both groups’ ALT activity was significantly lower than that in the S_0_ group (*p* < 0.05). The AST activity in the S_0.1_ group was significantly lower than that in the other two groups (*p* < 0.05). There was no significant difference in the TP content of serum among the three groups (*p* > 0.05). The ALB level in the S_0.2_ group was significantly higher than that in the S_0.1_ group but significantly lower than that in the S_0_ group (*p* < 0.05).

### 3.2. Levels of LZM, ACP, and AKP in Serum

The serum nonspecial immune indexes of the hybrid grouper are shown in Table 3. The enzyme activities of LZM, ACP, and AKP in the S_0_ group were not significantly different from those in the S_0.2_ group, while they were significantly lower than those in the S_0.1_ group (*p* < 0.05).

### 3.3. Liver Antioxidative Indexes

As shown in Table 4, the content of MDA in the S_0_ group was significantly higher than that in the experiment groups (*p* < 0.05). The SOD and CAT enzyme activities in the S_0.1_ group were significantly higher than those in the other groups (*p* < 0.05). The enzyme activity of GSH-PX in the S_0.2_ group was significantly higher than that in the S_0_ group but significantly lower than that in the S_0.1_ group (*p* < 0.05).

### 3.4. Transcriptome Analysis in S_0_ and S_0.1_ Group Fish Liver

**Figure 1:**
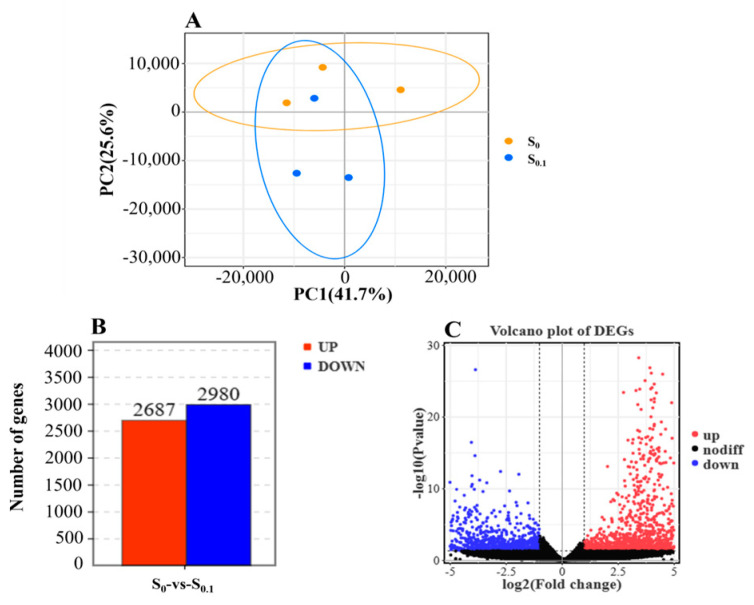
Transcriptome analysis in hybrid grouper liver. Note: (**A**) principal component analysis of livers in S_0_ and S_0.1_ (PCA); (**B**) number of up-regulated and down-regulated differentially expressed genes (DEGs); (**C**) volcano plot of DEGs between S_0_ group and S_0.1_ group.

To explore the potential mechanisms of steroidal saponins on glucose lipid metabolism in hybrid grouper liver, first, the high-throughput RNA-sequencing technique was used in this experiment. The goodness of reproducibility between samples within groups and the degree of variability of samples between groups were analyzed with PCA (Figure 1A). Then, differentially expressed genes (DEGs) were separated into the S_0_ group and the best-addition S_0.1_ group of steroidal saponins. A total of 5667 DEGs were identified under the log_2_^|Fold change|^ > 2.0 threshold and with a *p* value < 0.05 (Figure 1C), including 2687 up-regulated DEGs and 2980 down-regulated DEGs (Figure 1B).

### 3.5. Signaling Pathway Network and Key Genes of Glucose and Lipid Metabolism

**Figure 2:**
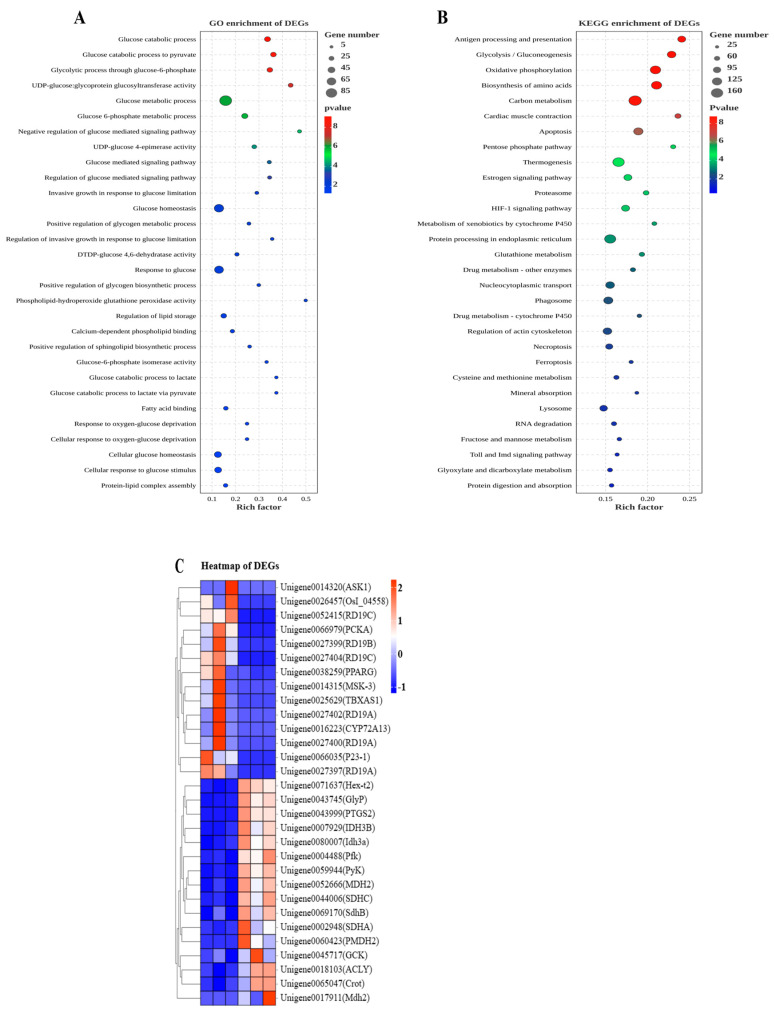
GO and KEGG enrichment of DEGs in fish liver. Note: (**A**) GO enrichment of DEGs; (**B**) KEGG enrichment of DEGs; (**C**) heatmap of DEGs.

The DEGs were entered into the GO and KEGG databases to search their involved signaling pathways (Figure 2). The present experiment involved 46 key different genes (Table 5), including 18 down-regulated and 28 up-regulated key genes. Of these, 15 genes were significantly down-regulated, and 24 genes were significantly up-regulated. Then, 30 DEGs were selected, as shown in Table 5, and clustered into a heatmap depending on their expression level and classification (Figure 2C).

The interesting DEGs were enriched in GO, focusing on the thirty items in this study. To be exact, among the 30 enrichment items analyzed using the rich factor value, 24 items were associated with glucose metabolism, including glycogen metabolism (positive regulation of glycogen mediated process, positive regulation of glycogen biosynthetic process, UDP-glucose-4-epimerase activity, and UDP-glucose: glycoprotein glycosyltransferase activity), glycolysis (glucose catabolic process, glucose catabolic process to pyruvate, glucose catabolic process to lactate, and glucose catabolic process to lactate via pyruvate), glycogenesis (glycolytic process through glucose-6-phosphate, glucose-6-phosphate metabolic process, and DTDP-glucose-4,6-dehydratase activity), tricarboxylic acid cycle (glucose-6-phosphate isomerase activity), glucose metabolic process, regulation of glucose-mediated signaling pathway, invasive growth in response to glucose limitation, negative regulation of glucose-mediated signaling pathway, glucose-mediated signaling pathway, glucose homeostasis, regulation of invasive growth in response to glucose limitation, response to glucose, response to oxygen glucose deprivation, cellular response to oxygen glucose deprivation, cellular glucose homeostasis, and cellular response to glucose stimulus (Figure 2A). Meanwhile, six items were related to lipid metabolism, including phospholipid-hydroperoxide glutathione peroxidase activity, regulation of lipid storage, calcium-dependent phospholipid binding, positive regulation of sphingolipid biosynthetic process, fatty acid binding, and protein–lipid complex assembly (Figure 2A).

In addition, the focused DEGs were enriched into 30 interesting KEGG signaling pathways. The 30 signaling pathways showed that glucose metabolism (glycolysis/gluconeogenesis, oxidative phosphorylation, carbon metabolism, pentose phosphate pathway, fructose and mannose metabolism, glyoxylate and dicarboxylate metabolism, and thermogenesis), amino acids metabolism (biosynthesis of amino acids, proteasome, protein processing in endoplasmic reticulum, glutathione metabolism, cysteine and methionine metabolism, and protein digestion and absorption), lipid metabolism (metabolism of xenobiotics by cytochrome P450 and drug metabolism by cytochrome P450), and other signaling pathways (antigen processing and presentation, cardiac muscle contraction, apoptosis, estrogen signaling pathway, HIF-1 signaling pathway, drug metabolism by other enzymes, nucleocytoplasmic transport, phagosome, regulation of actin cytoskeleton, necroptosis, ferroptosis, mineral absorption, lysosome, RNA degradation, and toll and imd signaling pathway) were mainly involved in this experiment (Figure 2B).

### 3.6. Metabolism Analysis in S_0_ and S_0.1_ Group Fish Liver

**Figure 3:**
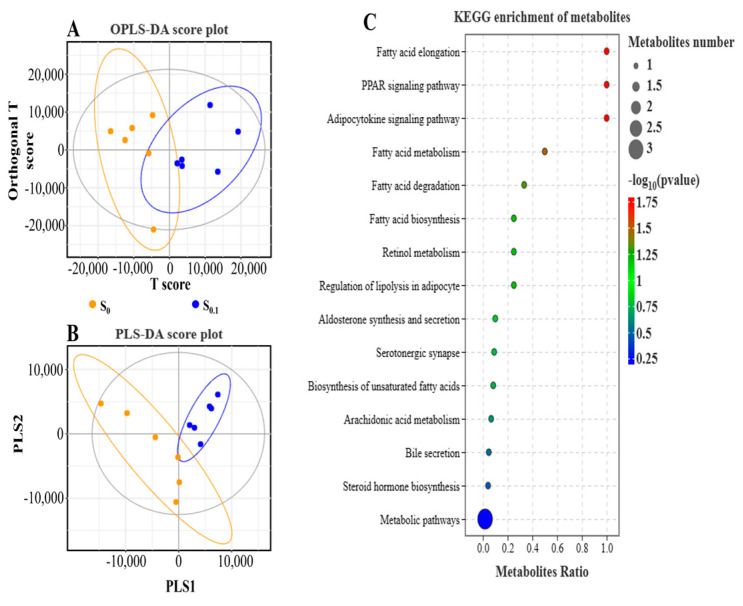
Metabolome analysis in hybrid grouper liver. Note: (**A**) the result of discriminant analysis of Orthogonal Projections to Latent Structures Discriminant Analysis (OPLS-DA); (**B**) identification analysis was performed with Partial Least Squares Discriminate Analysis (PLS-DA); (**C**) KEGG enrichment of metabolites.

After constructing the model with Orthogonal Projections to Latent Structures Discriminant Analysis (OPLS-DA) and Partial Least Squares Discriminate Analysis (PLS-DA), the sample clustering results were described using OPLS-DA and PLS-DA score plots (Figure 3A,B). It was shown that metabolites may be clustered depending on differences in the presence or absence of steroidal saponins in high-lipid diets. For example, the S_0.1_ group was located on the positive side of the T-score and Partial Least Squares-1 (PLS1). The metabolite products were identified in this research, including prostaglandin G2/H2, thromboxane-2, malate, phosphoenolpyruvic acid, and fructose-6-phosphate. Moreover, the metabolites were enriched into 15 KEGG signaling pathways (Figure 3C). The results showed that lipid metabolism (bile secretion, *ppar* signaling pathway, adipocytokine signaling pathway, regulation of lipolysis in adipocytes, fatty acid metabolism, fatty acid elongation, fatty acid degradation, fatty acid biosynthesis, biosynthesis of unsaturated fatty acids, arachidonic acid metabolism, and steroid hormone biosynthesis) and other metabolism pathways were mainly involved in this experiment.

In addition, thirty-two metabolites related to lipid metabolism are listed in Table 6, of which eight metabolites were significantly down-regulated, nine metabolites were significantly up-regulated, nine metabolites were down-regulated, and six metabolites were up-regulated. At the same time, there are eighteen key metabolites related to glucose metabolism listed in Table 6, of which three metabolites were significantly down-regulated, nine metabolites were down-regulated, two metabolites were significantly up-regulated, and four metabolites were up-regulated.

### 3.7. Combined Analysis of Transcriptomic and Metabolomic Data

**Figure 4:**
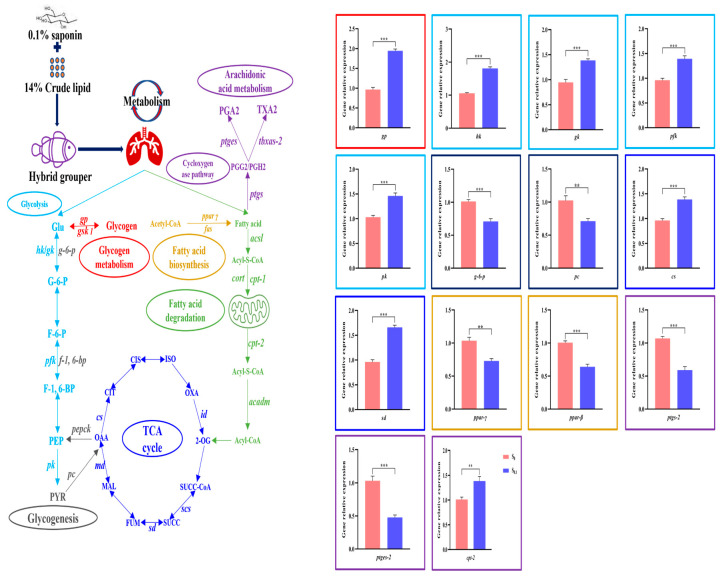
Combining transcriptome and metabolome analysis and quantitative reverse transcription PCR verification of DEGs in hybrid grouper liver glucose lipid metabolism. The dynamics of metabolites and gene expression are shown in the diagram above. The detailed DEGs and metabolite expression are shown in Table 3 and Table 4. Note: glycogen phosphorylase (*gp*), glycogen synthase kinase-1 (*gsk-1*), glucose (Glu), hexokinase (*hk*), glucokinase (*gk*), glucose-6-phosphatase (*g-6-p*), glucose-6-phosphate (G-6-P), fructose-6-phosphate (F-6-P), phosphofructokinase (*pfk*), fructose-1,6-bisphosphatase (*f-1,6-bp*), fructose-1,6-bisphosphate (F-1,6-BP), phosphoenolpyruvic acid (PEP), pyruvate kinase (*pk*), pyruvic acid (PYR), pyruvate carboxylase (*pc*), phosphoenolpyruvate carboxykinase (*pepck*), oxaloacetic acid (OAA), citrate synthase (*cs*), citrate (CIT), cis-aconitate (CIS), isocitrate (ISO), oxalosuccinate (OXA), isocitrate dehydrogenase (id), 2-oxoglutarate (2-OG), succinyl-CoA (SUC-CoA), succinyl-CoA synthetase (*scs*), succinic acid (SUCC), succinate dehydrogenase (*sd*), fumarate (FUM), malate (MAL), malate dehydrogenase (*md*), peroxisome proliferator-activated receptor γ (*ppar-γ*), fatty acid synthase (*fas*), prostaglandin-endoperoxide synthase (*ptgs*), prostaglandin G2/H2 (PGG2/PGH2), prostaglandin E synthase (*ptges*), prostaglandin A2 (PGA2), thromboxane-2 synthase (*tbxas-2*), thromboxane A2 (TXA2), acyl-CoA synthetase (*acsl*), carnitine acyltransferase 1 (*cpt-1*), carnitine acyltransferase 2 (*cpt-2*), and acyl-CoA dehydrogenase (*acadm*). For the comparison of the S_0_ group with the S_0.1_ group, the Student’s *t*-test was used in the experiment. Asterisks (** and ***) stand for significant differences with *p* < 0.01 and *p* < 0.001, respectively.

In an attempt to explore the changes in metabolites and mRNA transcripts during glycose lipid metabolism, we performed correlation analysis using transcriptomic and metabolomic data (Figure 4). It was shown that the addition of steroidal saponins to high-lipid diets contributed to activating glycogen breakdown, glycolysis, the TCA cycle, and the fatty acid β-oxidation pathway while inhibiting glycogen synthesis, glycogenesis, fatty acids biosynthesis, cyclooxygenase, and the arachidonic acid signaling pathway.

Next, 14 DEGs were randomly selected from the samples of the S_0_ and S_0.1_ groups. Then, they were examined using qRT-PCR to verify the reliability of the RNA-Seq data. The results of qRT-PCR and RNA-seq were consistent, as shown in Figure 4, indicating a reliable result of data analysis.

### 3.8. Gene Expression of ptgs-1, ptges-1, and tbxas-2

**Figure 5:**
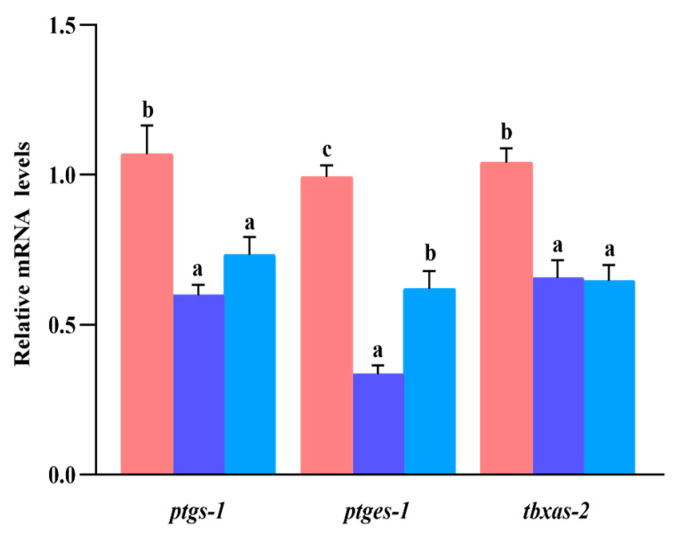
Efficacy of dietary steroidal saponins on arachidonic acid signaling pathway of experimental fish livers. Note: (a–c): Values from smallest to largest.

There was no significant difference in liver *ptgs-1* and *tbxas-2* mRNA expression between the S_0.1_ group and the S_0.2_ group, but both groups’ expression levels were significantly lower than those in the S_0_ group (*p* < 0.05) (Figure 5). In the S_0.2_ group, the expression of *ptges-1* mRNA was significantly higher than in the S_0.1_ group while significantly lower than in the S_0_ group (*p* < 0.05).

### 3.9. Gene Expression of Glucose Metabolism in Liver

**Figure 6:**
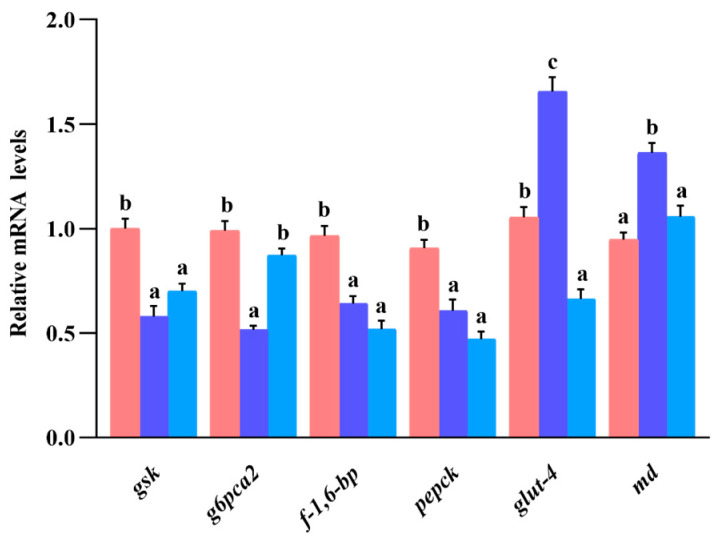
Efficacy of dietary steroidal saponins on glucose metabolism in hybrid grouper liver. Note: (a–c): Values from smallest to largest.

As indicated in Figure 6, the expression of *gsk*, *f-1*,*6-bp*, and *pepck* mRNA in the S_0.1_ group was not significantly different from that in the S_0.2_ group (*p* > 0.05), but the three genes’ expression levels were significantly lower in the S_0.1_ and S_0.2_ groups than in the S_0_ group (*p* < 0.05). The *g6pca2* mRNA expression level did not show differences between the S_0_ and S_0.2_ groups (*p* > 0.05), but it was significantly higher in the S_0.1_ group (*p* < 0.05). The expression of *glut-4* mRNA in the S_0_ group was significantly higher than that in the S_0.2_ group but significantly lower than that in the S_0.1_ group (*p* < 0.05). The *md* mRNA expression levels in the S_0_ group and S_0.2_ group were not found to be significantly different, while both groups’ levels were significantly lower than that in the S_0_ group (*p* < 0.05).

### 3.10. Gene Expression of Lipid Metabolism in Liver

**Figure 7:**
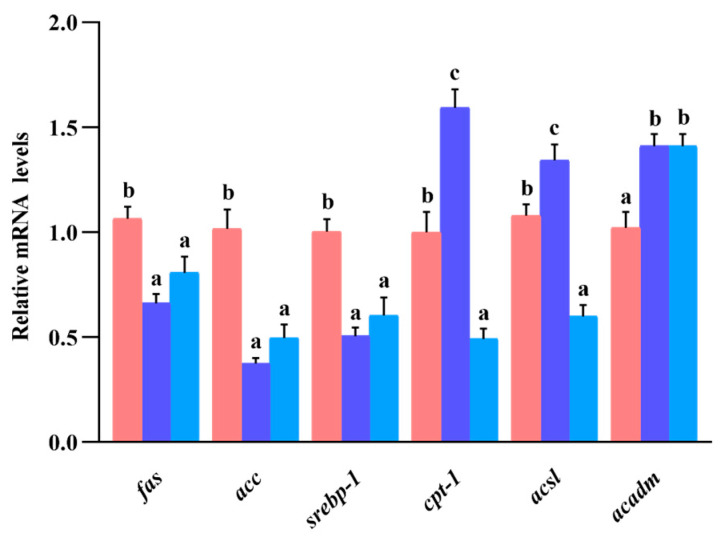
Effect of dietary steroidal saponins on lipid metabolism in the liver of hybrid grouper. Note: (a–c): Values from smallest to largest.

As can be seen in Figure 7, the *fas*, *acc*, and *srebp-1* mRNA expression levels between the S_0.1_ and S_0.2_ groups were not significantly different (*p* > 0.05), but they were significantly lower than those in the S_0_ group (*p* < 0.05). Although the expression of *cpt-1* and *acsl* mRNA in the S_0_ group was significantly higher than that in the S_0.2_ group, it was significantly lower than that in the S_0.1_ group (*p* < 0.05). The *acadm* mRNA expression in the S_0_ group was significantly lower than that in the S_0.1_ and S_0.2_ groups (*p* < 0.05).

## 4. Discussion

After ingestion of high-lipid diets (17% and 15% crude lipid), the area of lipid droplets in hybrid grouper liver increased, and the activities of the LZM, AKP, ACP, SOD, and CAT enzymes in the liver and serum decreased, while MDA, ALB, AST, and ALT levels were higher [7,27]. When high-lipid diets (15% crude lipid) were supplemented with saponins, the lipid droplet area decreased in the liver of hybrid grouper, and SOD, CAT, and GSH-PX activities significantly increased to enhance the antioxidant capacity of the fish [20]. In carp [17], white shrimp [28], and swimming crab (*Portunus trituberculatus*) [29], studies have demonstrated that saponins can decrease serum ALB, AST, and ALT enzyme activities and increase antioxidant and nonspecific immune enzyme activities in the liver to enhance the immune capacity of the body. A previous study demonstrated that 0.1% steroidal saponin significantly increased the activities and gene expression of SOD, CAT, GSH-PX, and GR in hybrid grouper liver and serum and decreased the MDA content [21]. In the present experiment, compared to the control group, supplementation of 0.1% steroidal saponins to a 14% crude lipid diet significantly increased LZM, ACP, and AKP enzyme activities and decreased ALB, ALT, and AST enzyme activities in the serum of hybrid grouper.

Previous studies on Chinese mitten crab (*Eriocheir sinensis*) [30] and olive flounder (*Paralichthys olivaceus*) [31] found that arachidonic acid can enhance the antioxidant capacity of the organism by increasing SOD, CAT, AKP, ACP, and LZM activities in the liver and serum. Three metabolic pathways—cytochrome P450, cyclooxygenase, and lipoxygenase—are all engaged in the metabolism of arachidonic acid [32], in which inhibition of the expression and content of key genes and metabolites of the cyclooxygenase metabolic pathway could improve the anti-inflammatory effects of the body [33]. Compared with the control group, the *sod*, *cat*, and *gsh-px* genes’ expression in hybrid grouper liver was up-regulated with the addition of 0.1% steroidal saponins, and the levels of PGG2/PGH2, PGA2 and TXA2 metabolites, *ptgs-1* and *ptgs-2*, *ptges-1* and *ptges-2*, and *tbxas-2* mRNA significantly decreased. Both KEGG and GO showed that these genes and metabolites were enriched in the cyclooxygenase metabolic pathway. The upstream *ptgs-1* and *ptgs-2* genes can activate the cyclooxygenase metabolic pathway to release the intermediate products PGG2 and PGH2, which stimulate the downstream *ptges-1*, *ptges-2*, and *tbxas-2* genes to produce the downstream products PGA2 and TXA2, mediating the inflammatory response [34,35]. The present study also demonstrated that the supplementation of 0.1% and 0.2% steroidal saponins in the diets contributed to a decrease in the expression of *ptgs-1* and *ptgs-2*, *ptges-1* and *ptges-2*, and *tbxas-2* mRNA in the liver of hybrid grouper, inhibited the expression of key genes in the cyclooxygenase metabolic pathway, and played an anti-inflammatory role in the body’s immune system. Research in mice demonstrated that saponins can down-regulate *ptgs-1* and *ptgs-2*, *ptges-1* and *ptges-2*, and *tbxas-2* mRNA expression in the liver and decrease the levels of PGG2, PGH2, PGA2, and TXA2 metabolites [36,37]. Consequently, steroid saponins can protect the liver by effectively removing lipid peroxidation products from the body by maintaining oxidative homeostasis through non-specific immune factors, thus improving the immunity of the fish and enabling the orderly metabolism of nutrients in the fish. However, compared to the S_0.2_ group, 0.1% steroidal saponin significantly decreased serum and liver ALB and AST enzyme activities and *ptges-1* gene expression and significantly increased LZM, ACP, and AKP enzyme activities. Research on Atlantic salmon (*Salmo salar* L.) [38] found that the dietary addition of 0.2% soya saponins increased serum SOD and CAT activities, but supplementation of 0.2% or 0.32% *soy* saponin decreased carnivorous field eels’ (*Monopterus albus*) [39] and Japanese flounder’s [40] antioxidant capacity. Research on omnivorous carp [41] found that antioxidant capacity was significantly inhibited when dietary *Momordica charantia* saponins were provided at levels above 0.64%. Thus, high doses of saponins are toxic to fish and decrease antioxidant capacity.

A previous study showed that supplementation of 0.1% steroidal saponins to high-lipid diets significantly increased the activity and gene expression of hexokinase, pyruvate kinase, and phosphofructokinase in the liver and significantly decreased glucose and glycogen content in the liver and serum of grouper [21]. Those phenomena indicated that steroid saponins had a potential role in stimulating carbohydrate utilization by the organism by elevating the activity and gene expression of the enzymes mentioned above. In this experiment, analysis of transcriptomic and metabolomic data showed that 0.1% steroidal saponins increased the expression of genes such as *gp*, *pk*, and *md* and metabolites such as G-6-P, F-6-P, and PEP in hybrid grouper liver, which were enriched in the glycolysis pathway, and decreased the expression of genes such as *gsk*, *g6pca2*, *f-1,6-bp*, and *pepck* and metabolite levels such as PYR and OAA in the gluconeogenesis pathway. It was suggested that steroidal saponins can inhibit the gluconeogenesis pathway by activating the glycolysis pathway and gradually reducing intermediate products, such as pyruvate, a product of glycolysis and a substrate of the gluconeogenesis pathway. At the same time, the ingestion of diets containing 0.1% and 0.2% steroidal saponins up-regulated *glut-4* and *md* gene expression in hybrid grouper liver, while down-regulating *gsk*, *g6pca2*, *f-1,6-bp*, and *pepck* mRNA expression, consistent with the analysis of omics data. An investigation on grouper and carp found that hepatic expression of *hk*, *gk*, and *pk* genes was up-regulated by saponins and down-regulated by *g-6-p*, *f-1,6-bp*, and *pepck* genes in the liver [42,43]. As a result, steroidal saponins can effectively promote the ability of hybrid grouper to utilize carbohydrates in the diet by activating the expression of genes and metabolite content in the glycolysis pathway while inhibiting the gluconeogenesis pathway.

The ingestion of high-lipid diets by grass carp (*Ctenopharyngodon idella*) (11% and 16% crude lipid) [44,45] and Nile tilapia (*Oreochromis niloticus*) (12% crude lipid) [46] caused an increase in serum and liver triglyceride and cholesterol levels. Studies on field eel [39], catfish (*heterobranchus longifilis*) [47], and largemouth bass (*Micropterus salmoides*) [48] revealed that saponin reduced *fas*, *acc*, *ppar-γ*, and *srebp-1* mRNA expression in the liver while elevating the gene expression of *cpt-1*, *cpt-2*, *acsl*, and *acadm*. In this experiment, supplementation with 0.1% steroidal saponins significantly reduced triglyceride and cholesterol levels in the liver and serum of hybrid grouper [21], decreased the expression of genes such as *fas* and *ppar-γ* and metabolite levels such as eicosapentaenoic acid and palmitic acid in the liver, thereby inhibiting fatty acid biosynthesis while elevating the fatty acid β-oxidation pathway in *cpt-1*, *cpt-2*, *acsl*, and *acadm* gene expression and metabolite levels such as carnitine and citric acid. Meanwhile, the mRNA expression levels of *fas*, *acc*, and *srebp-1* in the liver significantly decreased, while the gene expression levels of *cpt-1*, *acsl*, and *acadm* increased in the hybrid grouper who were fed a diet supplemented with 0.1% and 0.2% steroidal saponins. Steroid saponins can activate the fatty acid β-oxidation signaling pathway, increase the expression of key genes and metabolite levels, promote fatty acid transport to mitochondria, and, thus, reduce lipid deposition in grouper liver [49]. However, previous studies have found that saikosaponin alleviates hepatic steatosis in hybrid grouper by activating the AMPK/PPARα signaling pathway after the fish consume a high-lipid diet (15% crude fat) [20]. This was inconsistent with the findings of the present study. Saikosaponin belongs to the triterpenoid saponins, while our experimental steroid saponins belong to the steroid group of saponins. It was possible that the saponin species had different effects on hybrid grouper.

## 5. Conclusions

Compared with the control group, the 0.1% of steroidal saponins effectively scavenged lipid peroxidation products and enhanced the immune defense mechanism of fish by inhibiting the levels of metabolites and genes in the cyclooxygenase metabolic pathway. Further, 0.1% of steroidal saponins promoted more efficient utilization of carbohydrates and lipids in the diets by activating the expression of genes and metabolites levels in the glycolysis pathway and fatty acid β-oxidation pathway and inhibiting the gluconeogenesis pathway, facilitating the conversion and deposition of feed proteins in grouper.

## Figures and Tables

**Table 1:** Primer pair sequences for qRT-PCR.

Target Gene	Nucleotide Sequence (5′-3′)	Accession No.
Glycogen phosphorylase (*gp*)	F: TCTCCCGTGTTCTTTACCC R: GCCATTGCTGGATGAGTG	MT548584
Glycogen synthase kinase (*gsk*)	F: TACACTGCCTGACCAAGACC R: TAATGTGGCTGGAGACGAAT	MT548583
Hexokinase-1 (*hk*)	F: ACTTTGGGTGCAATCCTGAC R: AGACGACGCACTGTTTTGTG	BC067330.1
Glucokinase (*gk*)	F: TGGGTTTTACCTTCTCCTT R: AGTCCCCTCGTCTCTTGAT	MH213270
Phosphofructokinase (*pfk*)	F: AAACGCCCATGCAAACTAC R: CAACCTCTCTGACAGCCAC	MH213271
Pyruvate kinase (*pk*)	F: TCCTGGAGCATCTGTGTCTG R: GTCTGGCGATGTTCATTCCT	BC152219.1
Glucose-6-phosphatase (*g-6-p*)	F: GCCTGTGGATGCTAATGGG R: GGAGGTCAAGAAGAGAGTCGTG	MT548587
Glucose-6-phosphatase catalytic subunit 2 (*g6pca2*)	F: CACAGTCCGTCCTCACAT R: GCAAAACAGCGTCCATAA	MH213269
Fructose-1,6-bisphosphatase (*f-1,6-bp*)	F: ACAGTCTGAATGAAGGCTAC R: CTCATACAACAGCCTCAGCT	XM019099298.1
Pyruvate carboxylase (*pc*)	F: GTTGCCCACAACTTCAGCAAG R: ATGCTGGGCTG TGAAGTCATC	NM_022172.3
Phosphoenolpyruvate carboxykinase (*pepck*)	F: CGTGCTGGACTGGATGTTC R: CCAAGCCCTGGAGGTTCA	MT548586
Glucose transporter 4 (*glut-4*)	F: ATTCCTCTACATCGTCCGC R: CCATCTTCCTCTTCTCCTC	XM_010737169.3
Citrate synthase (*cs*)	F: GAAGGAACTCGGTGGTGAGG R: GACCATAGCCTGGCACAACT	BC165743.1
Succinate dehydrogenase (*sd*)	F: ACCTTGGACCTGCTGTGTTGATG R: GCGGTAGAGGGAGAACGGAT	SRP152627
Malate dehydrogenase (*md*)	F: ATGCCATGAATGGAAAGGAG R: TCCTCGCCCTTCTTGATAGA	XM_038711981.1
Prostaglandin-endoperoxide synthase 1 (*ptgs-1*)	F: AAGGTCTGGGTCACGGAGTG R: CAAGAGAATCTCCATAAATGTGTCCA	GENE-020530-1
Prostaglandin-endoperoxide synthase 2 (*ptgs-2*)	F: GAGTACTGGAAGCCGAGCAC R: GATATCACTGCCGCCTGAGT	AM296029
Prostaglandin E synthase 1 (*ptges-1*)	F: TAATAACGGGACAAGTGAGGC R: GGCAAACAAGTAGGCAACG	XM_010750942
Prostaglandin E synthase 2 (*ptges-2*)	F: TGGCTGTGTTTGGCGTCCTCA R: TCTTGGTGTTCTGCGGTGTCCT	XM_019272321
Thromboxane-2 synthase (*tbxas-2*)	F: AGCTGCATGATGGGATCTGTCAATC R: AGGTGGATGATGCGATGTGTGAATC	AY398422
Peroxisome proliferator-activated receptor *γ* (*ppar-γ*)	F: ACCGCAGCACGAAGAACAAC R: TGGACGCCATAGTGAAACCC	KM052849.1
Peroxisome proliferator-activated receptor β (*ppar-β*)	F: GCTACAGAGCAGCACGACA R: CTCCTCATCTTCGCTTTCC	DQ232867.1
Fatty acid synthase (*fas*)	F: GGCGGCATTGTAGGCATTA R: CAATCAAAGTGTAGCCTCGGTAG	FJ196231.1
Acetyl-CoA carboxylase (*acc*)	F: GAGAAGGCACCAGAAGATCATAG R: CACAGTACCTGCACTCACATAG	KX066238.1
Sterol regulatory element-binding protein 1 (*srebp-1*)	F: TGGAGTTTGGAGGACTGTTTG R: AAGATGAGGGTGGAGTTGGA	KU179485.1
Acyl-CoA synthetase (*acsl*)	F: CCATACACATCCACACCGAGT R: ACAGCTCTTCTATCTGGGGTC	GLEAN_10004723
Carnitine acyltransferase 1 (*cpt-1*)	F: TGCTCCACGGAAAGTGCTAC R: GCAGTGACCCTCCTCAGTGTAT	HM037343.1
Carnitine acyltransferase 2 (*cpt-2*)	F: CTATCTGGAGTGACATCATG R: CTCACTCACAGGTAGAGATG	XM019122007.1
Acyl-CoA dehydrogenase (*acadm*)	F: ACAAGGTTTTGAGGGCAGGT R: TTGGCACTAGCTTGAGCACT	NM_213010.2
*β-actin*	F: GGCTACTCCTTCACCACCACA R: TCTGGGCAACGGAACCTCT	AY510710.2

**Table 2:** Effects of dietary steroidal saponins on biochemical indexes of the hybrid grouper serum.

Indexes	S_0_	S_0.1_	S_0.2_
ALT/(U/L)	66.92 ± 2.29 ^b^	45.32 ± 1.05 ^a^	45.34 ± 1.71 ^a^
AST/(U/L)	8.02 ± 0.28 ^b^	6.32 ± 0.22 ^a^	7.55 ± 0.18 ^b^
TP/(mg/mL)	3.24 ± 0.14	3.84 ± 0.18	3.26 ± 0.23
ALB/(g/L)	52.05 ± 0.68 ^c^	29.72 ± 0.31 ^a^	35.89 ± 0.29 ^b^

Note: Values range from minimum to maximum. There are significant differences indicated with different letters (*p* < 0.05).

**Table 3:** Effects of dietary steroidal saponins on nonspecial immune indexes of fish serum.

Indexes	S_0_	S_0.1_	S_0.2_
LZM/(U/mL)	48.97 ± 4.48 ^a^	77.81 ± 4.38 ^b^	52.69 ± 1.25 ^a^
ACP/(King’s unit/L)	3.60 ± 0.05 ^a^	5.96 ± 0.10 ^b^	4.36 ± 0.18 ^a^
AKP/(King’s unit/L)	3.04 ± 0.11 ^a^	4.66 ± 0.11 ^b^	3.22 ± 0.12 ^a^

Note: Values range from minimum to maximum. There are significant differences indicated with different letters (*p* < 0.05).

**Table 4:** Efficacy of dietary steroidal saponins on antioxidative indexes of hybrid grouper livers.

Indexes	S_0_	S_0.1_	S_0.2_
MDA/(nmol/mgprot)	3.95 ± 0.27 ^b^	3.24 ± 0.11 ^a^	4.24 ± 0.21 ^a^
SOD/(U/mgprot)	137.35 ± 2.65 ^a^	164.22 ± 1.73 ^b^	133.57 ± 1.98 ^a^
CAT/(U/mgprot)	1.68 ± 0.06 ^a^	3.43 ± 0.15 ^b^	2.11 ± 0.04 ^a^
GSH-PX/(U)	4.73 ± 0.09 ^a^	7.61 ± 0.04 ^c^	6.54 ± 0.18 ^b^

Note: Values range from minimum to maximum. There are significant differences indicated with different letters (*p* < 0.05).

**Table 5:** Different expressions of DEGs related to glucose lipid metabolism in liver transcriptome of hybrid grouper.

Gene Symbol	Related Description	Gene ID	Log_2_^FC^	*p* Value
*pepck*	phosphoenolpyruvate carboxykinase	Unigene0066979	−11.53	0.00
*tbxas-2*	thromboxane synthase	Unigene0016223	−11.32	0.00
*scs*	succinyl-CoA synthetase	Unigene0027404	−11.21	0.00
*scs*	succinyl-CoA synthetase	Unigene0027402	−10.94	0.00
*scs*	succinyl-CoA synthetase	Unigene0027400	−10.49	0.00
*tbxas-2*	thromboxane synthase	Unigene0025629	−10.16	0.01
*ptges*	prostaglandin E synthase	Unigene0066035	−10.01	0.00
*scs*	succinyl-CoA synthetase	Unigene0052415	−9.90	0.00
*gsk-1*	glycogen synthase kinase	Unigene0014315	−9.72	0.03
*scs*	succinyl-CoA synthetase	Unigene0027399	−9.67	0.00
*scs*	succinyl-CoA synthetase	Unigene0027397	−9.58	0.00
*f-1,6-bp*	fructose-1,6-bisphosphatase	Unigene0026457	−9.22	0.04
*gsk-1*	glycogen synthase kinase	Unigene0014320	−9.04	0.02
*fas*	fatty acid synthase	Unigene0053299	−8.94	0.11
*ppar-γ*	peroxisome proliferator-activated receptor gamma	Unigene0038259	−1.20	0.00
*f-1,6-bp*	fructose-1,6-bisphosphatase	Unigene0086471	−0.75	0.01
*pc*	pyruvate carboxylase	Unigene0012223	−0.55	0.13
*g-6-p*	glucose-6-phosphatase	Unigene0029240	−0.38	0.28
*cpt-1*	carnitine palmitoyltransferase 1	Unigene0055448	0.77	0.08
*gk*	glucokinase	Unigene0045717	1.52	0.00
*cpt-2*	carnitine palmitoyltransferase 2	Unigene0047890	2.06	0.21
*cs*	citrate synthase	Unigene0018103	2.14	0.00
*sd*	succinate dehydrogenase	Unigene0069170	2.16	0.03
*acadm*	acyl-CoA dehydrogenase	Unigene0074561	2.16	0.03
*sod-2*	superoxide dismutase 2	Unigene0077359	2.34	0.00
*sd*	succinate dehydrogenase	Unigene0044006	2.90	0.03
*md*	malate dehydrogenase	Unigene0052666	2.90	0.00
*cort*	carnitine	Unigene0065047	3.08	0.03
*id*	isocitrate dehydrogenase	Unigene0080007	3.72	0.00
*pfk*	phosphofructokinase	Unigene0004488	3.79	0.03
*pk*	pyruvate kinase	Unigene0059944	3.88	0.00
*sd*	succinate dehydrogenase	Unigene0002948	4.39	0.01
*gp*	glycogen phosphorylase	Unigene0043745	4.46	0.00
*hk*	hexokinase	Unigene0071637	4.88	0.00
*id*	isocitrate dehydrogenase	Unigene0007929	5.21	0.00
*gsh*	glutathione peroxidase	Unigene0004325	9.52	0.37
*md*	malate dehydrogenase	Unigene0060423	9.57	0.01
*ptgs-2*	prostaglandin G/H synthase	Unigene0043998	9.60	0.03
*md*	malate dehydrogenase	Unigene0017911	10.03	0.03
*id*	isocitrate dehydrogenase	Unigene0022151	10.11	0.04
*acadm*	acyl-CoA dehydrogenase	Unigene0028577	10.21	0.01
*cat*	catalase	Unigene0028584	10.35	0.08
*acsl*	very long-chain-specific acyl-CoA dehydrogenase	Unigene0071297	10.38	0.03
*acadm*	acyl-CoA dehydrogenase	Unigene0027315	10.41	0.02
*ptgs-1*	prostaglandin G/H synthase	Unigene0043999	10.48	0.00
*sod-1*	superoxide dismutase 1	Unigene0052377	10.67	0.00

**Table 6:** Diverse levels of metabolites related to glucose lipid metabolism in liver metabolomic of hybrid grouper.

Metabolite Name	Metabolite Index	Log_2_^FC^	*p* Value
Lipid metabolism			
Methyl palmitate	Com_5883_pos	−2.18	0.00
Pentadecanoic acid	Com_12401_pos	−1.95	0.00
11(*Z*),14(Z),17(*Zz*)-Eicosatrienoic acid	Com_7643_pos	−1.87	0.00
11-Deoxy prostaglandin F1β	Com_422_pos	−0.93	0.01
Cytidine 5′-diphosphocholine	Com_1078_pos	−0.40	0.04
Lipoamide	Com_4536_pos	−0.39	0.02
Prostaglandin H1	Com_5241_neg	−0.36	0.53
15(*R*)-15-Methyl prostaglandin A2	Com_2092_pos	−0.34	0.04
Pyridoxal 5′-phosphate hydrate	Com_5949_pos	−0.28	0.04
Docosapentaenoic acid	Com_21_pos	−0.23	0.18
Prostaglandin F2α-1-glyceryl ester	Com_3467_pos	−0.2	0.26
7*Z*,10*Z*,13*Z*,16*Z*,19*Z*-docosapentaenoic acid	Com_11503_pos	−0.2	0.73
Thromboxane A2	Com_7080_pos	−0.1	0.73
all-*cis*-4,7,10,13,16-Docosapentaenoic acid	Com_778_pos	−0.09	0.67
d-threo-Isocitric acid	Com_12240_pos	−0.09	0.81
Glycerol-3-phosphate	Com_483_neg	−0.07	0.83
Prostaglandin A1 ethyl ester	Com_1353_neg	−0.01	0.95
Arachidonic acid	Com_22_neg	0.00	0.99
Citric acid	Com_168_neg	0.06	0.94
Palmitoyl sphingomyelin	Com_1814_pos	0.17	0.54
Levalbuterol	Com_11294_pos	0.24	0.66
1-Palmitoylglycerol	Com_10_pos	0.25	0.40
l-Palmitoyl carnitine	Com_13685_pos	0.38	0.27
*N*-Palmitoyl taurine	Com_3290_pos	0.45	0.01
2-Arachidonyl Glycerol ether	Com_1450_pos	0.56	0.00
Myristic acid	Com_8920_pos	1.10	0.00
Oleamide	Com_1_pos	1.25	0.03
12-Oxo phytodienoic acid	Com_669_pos	1.46	0.03
4-acetyl-4-(ethoxycarbonyl)heptanedioic acid	Com_2516_pos	2.31	0.00
*N*-Tetradecanamide	Com_3122_pos	2.99	0.00
(*R*)-3-Hydroxy myristic acid	Com_2141_neg	3.19	0.00
Hexadecanamide	Com_148_pos	3.66	0.00
Glucose metabolism			
Phenyl pyruvic acid	Com_735_neg	−1.28	0.45
Guanosine5′-diphosphate (GDP)	Com_2804_neg	−0.91	0.02
Trehalose-6-phosphate	Com_12250_pos	−0.63	0.33
Hydroxyglutaric acid	Com_28_neg	−0.60	0.02
Phosphoenolpyruvic acid	Com_1634_neg	−0.6	0.35
Citraconic acid	Com_2432_pos	−0.52	0.14
d-Galactosamine	Com_904_pos	−0.33	0.03
Malic acid	Com_82_neg	−0.20	0.49
Uridine 5′-monophosphate	Com_2039_pos	−0.15	0.46
Glycerol-3-phosphate	Com_177_neg	−0.15	0.68
Fumaric acid	Com_322_neg	−0.15	0.51
d-Glucose-1-phosphate	Com_6290_pos	−0.02	0.96
d-Glucose-6-phosphate	Com_237_neg	0.10	0.74
*N*-Acetyl-d-glucosamine 6-phosphate	Com_7268_neg	0.11	0.64
Glycerol-1-hexadecanoate	Com_1112_pos	0.29	0.04
Benzyl 6-*O*-beta-d-glucopyranosyl-beta-d-glucopyranoside	Com_15594_pos	0.34	0.04
6-phospho-d-glucono-1,5-lactone	Com_4851_pos	0.34	0.47
d-Glucosamine-6-phosphate	Com_10302_neg	0.95	0.52

## Data Availability

The data presented in this study are available in the main article.

## Supplementary Materials

The following supporting information can be downloaded at: https://www.mdpi.com/article/10.3390/ani13182894/s1, Table S1: Ingredients (g/100 g diet) and proximate composition (% dry matter), Table S2: The growth performance of hybrid groupers.
