# Supplementary material for: Integrated Metabolome and Transcriptome Analyses Reveal the Efficacy of Steroidal Saponins for Glucose and Lipid Metabolism in Hybrid Grouper (♀Epinephelus fuscoguttatus × ♂Epinephelus lanceolatu) Fed Higher-Lipid Diets
Source: Animals (Basel). 2023 Sep 12;13(18):2894. doi: 10.3390/ani13182894 (PMC10525917; doi:10.3390/ani13182894)
Supplement: Supplementary file 1 [file animals-13-02894-s001.zip › animals-2548263-supplementary.pdf]

Table S1. Ingredients (g/100 g diet) and proximate composition (% dry matter).

Ingredients	S_0	$S_{0.1}$	$S_{0.2}$
Fish meal	36.00	36.00	36.00
Poultry by-product meal	10.50	10.50	10.50
Soybean meal	6.00	6.00	6.00
Concentrated cottonseed protein	19.00	19.00	19.00
Wheat flour	16.00	16.00	16.00
Fish oil	4.25	4.25	4.25
Soybean oil	4.25	4.25	4.25
Choline chloride	0.50	0.50	0.50
$Ca(H_2PO_4)_2$	1.50	1.50	1.50
Vitamin C	0.05	0.05	0.05
Vitamin mix	0.50	0.50	0.50
Mineral mix	0.50	0.50	0.50
Betaine	0.50	0.50	0.50
Ethoxyquin	0.10	0.10	0.10
Steroid saponins	0.00	0.1	0.2
Microcrystalline cellulose	0.35	0.25	0.15
Total	100.00	100.00	100.00
Proximate analysis (%)			
Moisture	11.50	10.41	10.97
Crude protein	52.48	52.85	52.49
Crude lipid	13.93	13.62	13.85
Crude ash	11.74	11.96	11.92
Gross energy (KJ g^{-1} DM)	20.52	20.89	20.36

Table S2. The growth performance of hybrid groupers.

Items	S_0	$S_{0.1}$	$S_{0.2}$
survival rate /%	94.67 ± 6.11	96.00 ± 0.00	89.33 ± 2.31
percent weight gain /%	351.50 ± 12.75 ^{b}	353.96 ± 11.80 ^{b}	323.72 ± 7.37 ^{a}
specific growth rate (%/d)	2.69 ± 0.06 ^{b}	2.70 ± 0.05 ^{b}	2.58 ± 0.03 ^{a}
protein efficiency ratio	1.77 ± 0.10 ^{b}	1.81 ± 0.06 ^{b}	1.61 ± 0.02 ^{a}
protein deposition rate /%	31.57 ± 0.41 ^{b}	31.83 ± 1.05 ^{b}	27.9 ± 1.36 ^{a}
feed conversion ratio	1.05 ± 0.01	1.02 ± 0.02	1.03 ± 0.01
Feeding rate (%BW/d)	2.45 ± 0.15	2.40 ± 0.04	2.58 ± 0.06

Note: Values range from minimum to maximum. There are significant differences indicated with different letters ($p < 0.05$).
